# Investigation of the solubility and the potentials for purification of serum amyloid A (SAA) from equine acute phase serum – a pilot study

**DOI:** 10.1186/1756-0500-6-152

**Published:** 2013-04-16

**Authors:** Michelle B Christensen, Jens Christian Sørensen, Stine Jacobsen, Mads Kjelgaard-Hansen

**Affiliations:** 1 Department of Veterinary Clinical and Animal Sciences, University of Copenhagen, Groennegaardsvej 3, ground floor, Frederiksberg C 1870, Denmark; 2 Department of Food Science, University of Copenhagen, Thorvaldsensvej 40, 5th floor, Frederiksberg C 1870, Denmark; 3 Department of Large Animal Science, University of Copenhagen, Hoejbakkegaard Allè 5, Taastrup 2630, Denmark

**Keywords:** Biochemical properties, Serum amyloid A, Horse

## Abstract

**Background:**

Serum amyloid A (SAA) is useful as a diagnostic marker of systemic inflammation in horses, but only heterologous assays based on non-equine calibration and standardization are available for measurements of equine SAA. More accurate measurements could be obtained using purified species-specific SAA in native conformation for assay calibration and standardization. Further knowledge about the biochemical properties of SAA would facilitate a future production of native species-specific calibration material Therefore, the aim of the study was an investigation of the solubility and potentials for purification of equine SAA based on biochemical properties.

Freeze dried equine acute phase serum was dissolved in 70% 2-propanol, 8 M urea, and milli-Q water, respectively. Supercritical fluid extraction (SFE), size-exclusive chromatography (FPLC-SEC), and preparative isoelectric focusing (IEF) were performed in the attempt to purify. Immunostaining of IEF blots were used for isoform-specific detection of SAA in the preparations and purity was assessed by silverstained SDS-PAGE.

**Findings:**

SAA was soluble in 70% 2-propanol, 8 M urea and Milli-Q water. SAA was not separated in the lipophilic or ampipathic fractions following SFE. SAA was included in a FPLC-SEC-fraction of 237 kDa, despite the molecular weight known to be much smaller, suggesting binding to other serum constituents. SAA precipitated following separation of other serum proteins by preparative IEF.

**Discussion:**

No effective purification of SAA was achieved in the present study, but findings important for future investigations were made. The study suggested that SAA is not exclusively hydrophobic, but appears less hydrophobic when interacting with other serum components. These results suggest more complex aspects of solubility than previously believed, and indicate potentials for purification of native SAA.

## Findings

### Background

The acute phase response is the unspecific reaction to different stimulations causing systemic inflammation, like infection or trauma, and is accompanied by marked increases in the concentrations of major acute phase proteins [1]. Serum amyloid A (SAA) is a major acute phase protein in several species of interest in veterinary medicine [1,2], including horses [3,4], and is a useful diagnostic marker of inflammation across species [1]. SAA is an apolipoprotein of 10–15 kDa with amphipathic properties and at least three isoforms characterized by different isoelectric points have been detected in equine serum [5,6]. When released into the bloodstream, it tightly associates with high density lipoproteins (HDL) [7,8], thus displacing apolipoprotein A1 [9].

Purification of SAA may be of interest for several purposes such as molecular investigations of SAA [10,11], investigation of the kinetics [5] and functions of SAA [12], investigation of the pathogenesis of amyloidosis [13], or production of standards for SAA assays [14]. However, SAA tends to precipitate [13] or form large protein aggregates [15] when it is separated from HDL, and consequently, large-scale purification of SAA is a challenging discipline [16]. Several different protocols have been used in previous purifications of SAA across several different species, and some protocols have even been proposed to be useful for large-scale purification [13]. Ultra-centrifugation is a useful method for separation of lipoproteins [17], and ultracentrifugation and hydrophobic interaction chromatography [18] have both been used as important initial steps in several protocols for SAA purification, including a protocol for purification of equine SAA [5]. When such procedures are followed by additional purification steps such as gel-filtering, SAA purities of up to 98% have been demonstrated [18]. However, the usefulness of the purified products depends on the aim of the purification. The antigenic properties of SAA can, thus, be affected by some purification procedures [14], and immuno-based SAA assays are, consequently, often based on calibration and standards consisting of recombinant SAA [19], heterologous SAA [20], or pooled acute phase serum [14,21], rather than being based on purified species-specific SAA. Even though such heterologous calibrated assays are commercial available for diagnostic measurements of equine SAA [22-24], species-specific calibration material, consisting of the native purified protein is, however, needed to obtain precise measurements of SAA across different analytical methods and different laboratorial settings [23,25,26]. However, such native protein material is currently not commercially available [22].

Based on the known biochemical properties of SAA, size-exclusive chromatography (SEC) and preparative isoelectric focusing (IEF) have been used as main procedures in previous purifications [5,13,27], but because of the lipophilic nature of SAA, and the tight association to HDL, previous purifications have always been preceded by delipidation steps [5,27,28] like ultracentrifugation [17] or hydrophobic interaction chromatography (HIC) [18].

A recent report has suggested that porcine SAA has less lipophilic properties than previously believed [29]. If this is also true for other species, the general approach to SAA purification should probably be altered and large scale purification of SAA could maybe be possible without initial denaturing delipidation or other steps which could potentially affect the antigenic properties of the purified product. The use of more gentle protocols for SAA purification could potentially facilitate a commercial production of species specific calibration material for veterinary SAA assays, but further knowledge about the biochemical properties of SAA will be needed before such protocols can be established.

The aim of the study was, therefore, to investigate the solubility as part of the biochemical properties of equine SAA and the potentials for purification of SAA using size-exclusion chromatography (SEC) and preparative isoelectric focusing (IEF) without initial delipidation.

## Methods

**Figure 1:**
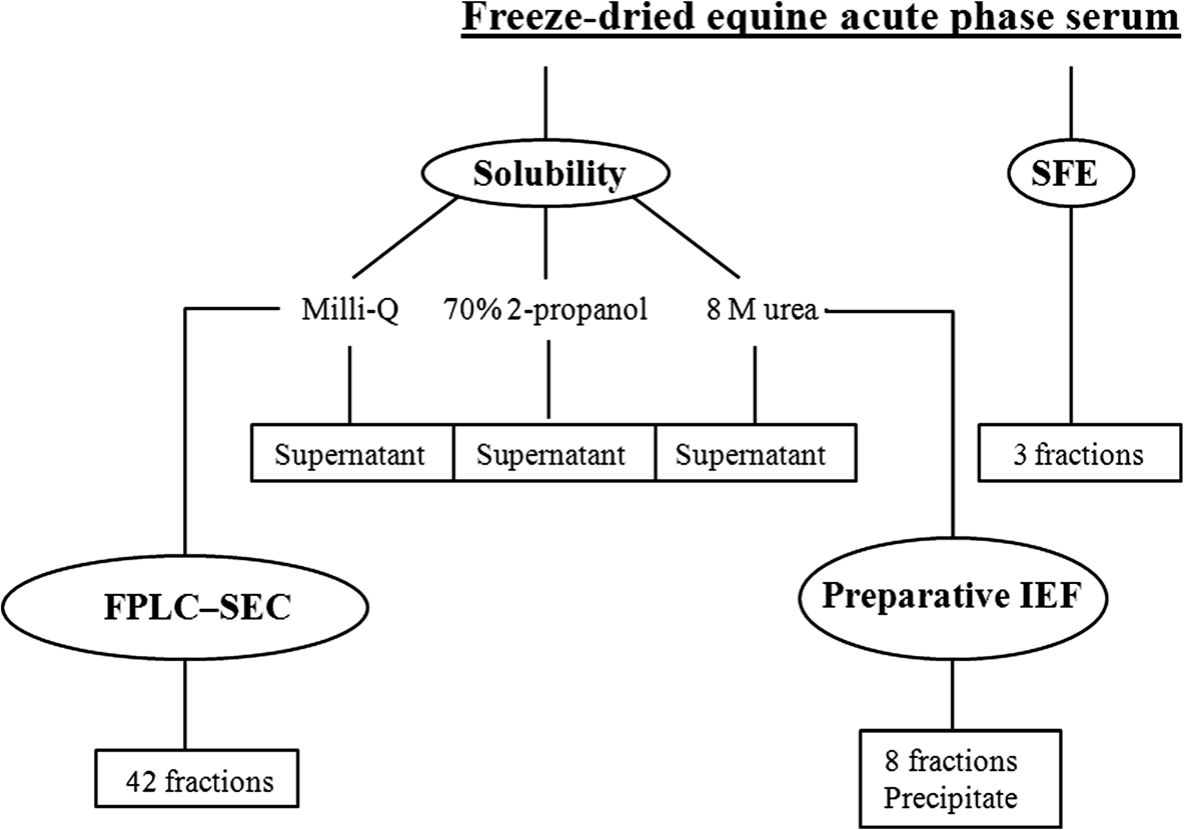
**Overview of the procedures included in the study.** Applied methods (encircled) and obtained products analyzed for SAA content and impurities (outlined in boxes) in the attempt to purify equine serum amyloid A (SAA). Isoforms of SAA were detected in supernatants, fractions, and precipitate using isoelectric focusing, electroblotting, and immunostaining. Purity of detected SAA was assessed by sodium dodecyl sulphate polyacryl amide gel electrophoresis (SDS-PAGE), followed by staining of serum proteins with silver nitrate. SFE: Supercritical fluid extraction with CO_2_. FPLC-SEC: Fast polymer liquid chromatography size-exclusive chromatography. IEF: Isoelectric focusing.

Pooled equine acute phase serum was used in the study, based on individual samples from client owned horses remaining after diagnostic analyses at the Central Laboratory, Department of Veterinary Clinical and Animal Sciences, University of Copenhagen, Denmark. The inclusion of samples in the present study was approved by the local ethical committee at the Department of Veterinary Clinical and Animal Sciences, University of Copenhagen, Denmark. The concentration of SAA in the serum pool was measured to 1600 mg/L, using a commercial available turbidimetric immunoassay previously validated for diagnostic measurements of equine SAA by members of our group [24]. Fifty millilitres of the serum pool was freeze dried from –4°C to room temperature at 10 mbar. An overview of the analytical and preparative procedures is given in Figure 1. The presence and composition of SAA was detected in serum and preparations by IEF, electroblotting (Amersham Pharmacia Biotech), and immunostaining with anti-SAA antibodies (Tri-delta Development Ltd), as previously done in several studies of SAA executed by our group [6,30,31].

Freeze dried serum was suspended in 70% 2-propanol, 8 M urea, and Milli-Q water, respectively, in a concentration of 10 mg freeze dried serum per milliliter solvent. Supernatants were analyzed for presence of dissolved SAA isoforms.

Supercritical fluid extraction (SFE) was performed using 600 mg freeze-dried serum in a Speed SFE system (Applied Separations). The extraction was performed at 50 mPa with a flow of 4 L CO_2_ per minute at 40°C for 30 minutes. Extracts were collected in glass vials and the extraction was repeated using 96% ethanol as modifier (1.0 mL/min). Extracts and remains were air dried, diluted in 8 M urea, and analyzed for the presence of SAA.

SEC was performed by fast polymer liquid chromatography (FPLC-SEC) using a Superdex™75 10/300 GL column (GE Healthcare) and a buffer containing 20 mM sodium dihydrophosphate and 50 mM sodium chloride, pH 6.9. A gel filtration standard containing thyroglobulin (660 kDa), gammaglobin (158 kDa), ovalbumin (44 kDa), myoglobin (17 kDa), and cyanocobalamin (1.35 kDa) (Bio-Rad) was used to make a standard curve for estimating molecular weights of different FPLC-fractions. Two-hundred microliter of the filtered supernatant resulting from the suspension of freeze-dried serum in Milli-Q water (described above) was injected to the column and separated at a flow of 1 ml/min yelding a pressure of 1.5 mPa. The molecular weight of equine SAA was verified by Sodium dodecyl sulphate – polyacrylamide gel electrophoresis (SDS-PAGE) in Phast Gel System (Amersham Pharmacia Biotech) followed by electroblotting and immunostaining as described above. A molecular weight marker (Bio Rad) was separated prior to blotting and stained with silvernitrate (Pharmacia LKB Biotechnology) [32] and used as standard.

Preparative IEF was performed in Hoefer IsoPrime IEF Purification Unit, (Amersham Pharmacia Biotech) [33,34]. Seven buffered polymembranes with pH 5, 6, 7, 7.5, 8, 8.5, and 9 were prepared with acrylamidobuffers (Fluka BioChemika and GE Healthcare Biosciences), in gels consisting of 30% acrylamide/bis-acrylamide (Sigma Aldrich), Tetramethylethylenediamine (Pharmacia Biothech) and 40% ammonium persulphate (Bio-Rad). Whatman glass microfiber filters GF/D 47 mm were encased in the membranes. Chambers were filled with Milli-Q water according to recommendations from the manufacturer, and 5 ml of the supernatant resulting from the suspension of freeze dried serum in 8 M urea (described above) was applied in separation chamber 2 (between the gels with pH 5 and 6). Separation was performed over 24 hours at a constant power of 4 W. Preparations were air dried and dissolved in 8 M urea prior to SAA detection. Visible precipitates were solubilized in 8 M urea overnight at room temperature, and the solute was investigated for presence of SAA.

Serum proteins in supernatants, fractions, and precipitate were stained with silvernitrate [32] following SDS-PAGE in Phast Gel System (Amersham Pharmacia Biotech) to assess purity of preparations, and a molecular weight marker was used as standard (Bio Rad).

## Results

**Figure 2:**
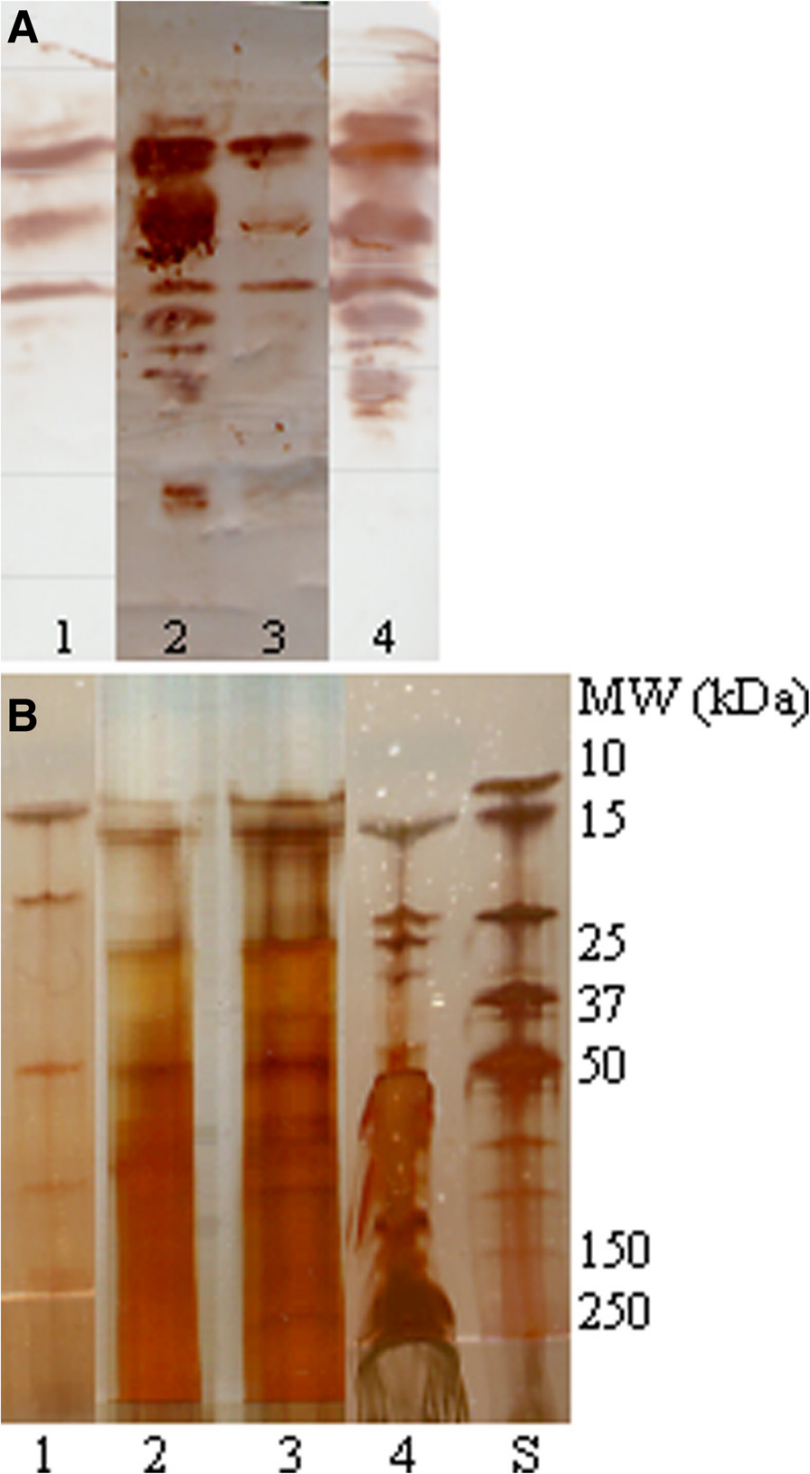
**Solubility of serum amyloid A.** Detection of serum amyloid A (SAA) isoforms (by isoelectric focusing and immunostaining) (**A**) and assessment of presence of non-SAA impurities in each of the dissolutions analyzed in A (by sodium dodecyl sulphate polyacryl amide gel electrophoresis and silverstaining) (**B**) following dissolution of freeze-dried equine acute phase serum in 70% 2-propanol (lane 1), 8 M urea (lane 2), and Milli-Q water (lane 3). SAA isoform pattern (**A**) and protein content (**B**) in untreated equine serum is seen in lane 4. The molecular weights (MW) of the BioRad standard are given to the right (S). SAA isoforms were detected in all solutes (**A**), but the most preserved isoform pattern of SAA was observed after dissolution of freeze dried serum in 8 M urea (lane 2).

Equine SAA was detected in supernatants of freeze dried serum solubilized in 70% 2-propanol, 8 M urea, and Milli-Q water. At least 3 isoforms of SAA were detected in each solvent, but the isoform pattern in serum dissolved in 8 M urea resembled the pattern of isoforms detected in untreated equine serum most accurately (Figure 2).

**Figure 3:**
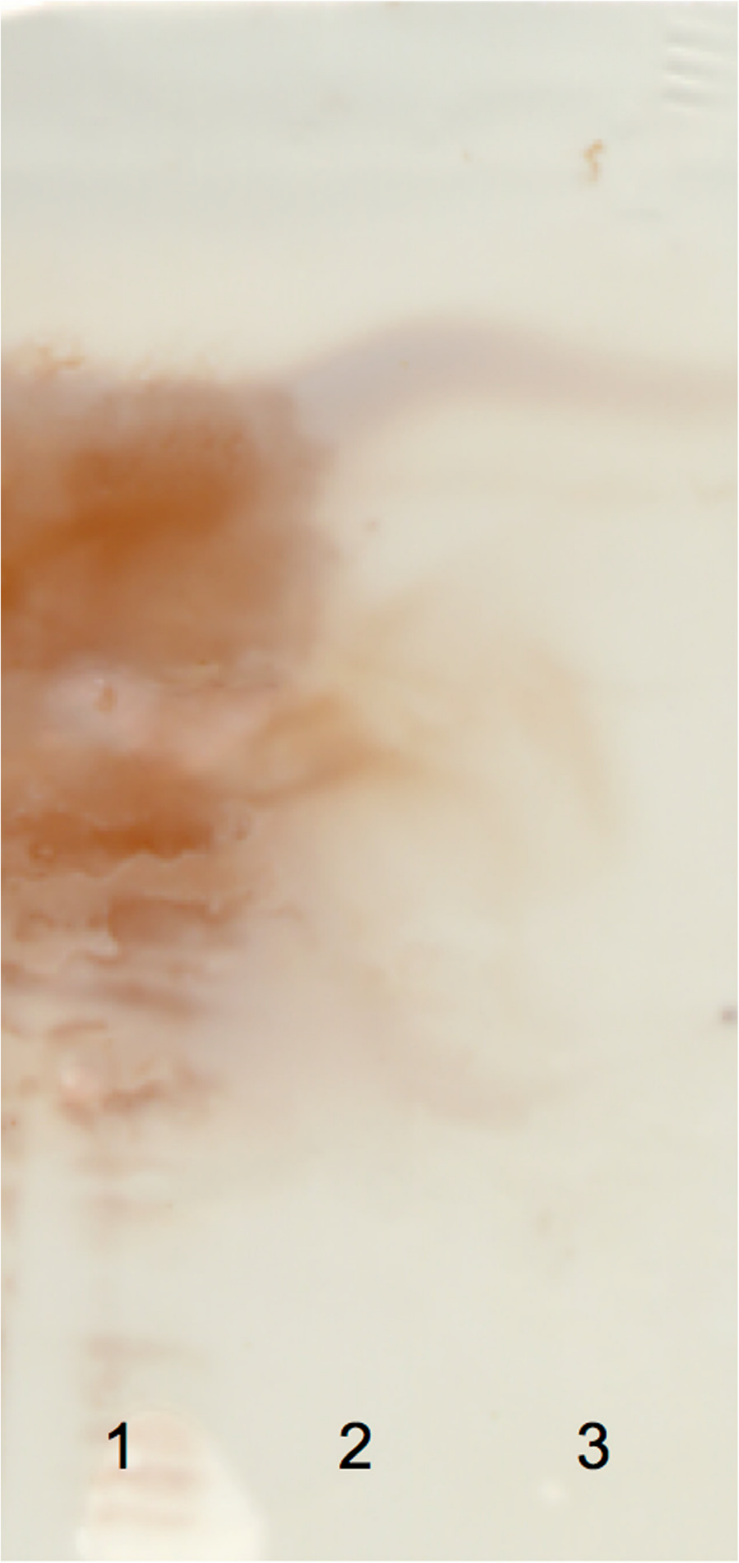
**No extraction of serum amyloid A by supercritical fluid extraction.** Serum amyloid A (SAA) detected by immunostaining after isoelectric focusing of fractions obtained after supercritical fluid extraction (SFE) of freeze dried equine acute phase serum. SAA was detected in the non-SFE-extractable fraction (lane 1), while no SAA was detected in the ampophilic/lipophilic (lane 2, extracts in CO_2_ modified by 96% ethanol, 1.0 mL/min) and the lipophilic fractions (lane 3, extracts in pure CO_2_).

Three fractions were obtained by SFE (Figure 1); a lipophilic fraction (extracted in pure CO_2_), a lipophilic/ampophilic fraction (extracted in CO_2_ modified with ethanol), and a non SFE-extractable fraction (remains). SAA was only detectable in the non SFE-extractable fraction (Figure 3).

**Figure 4:**
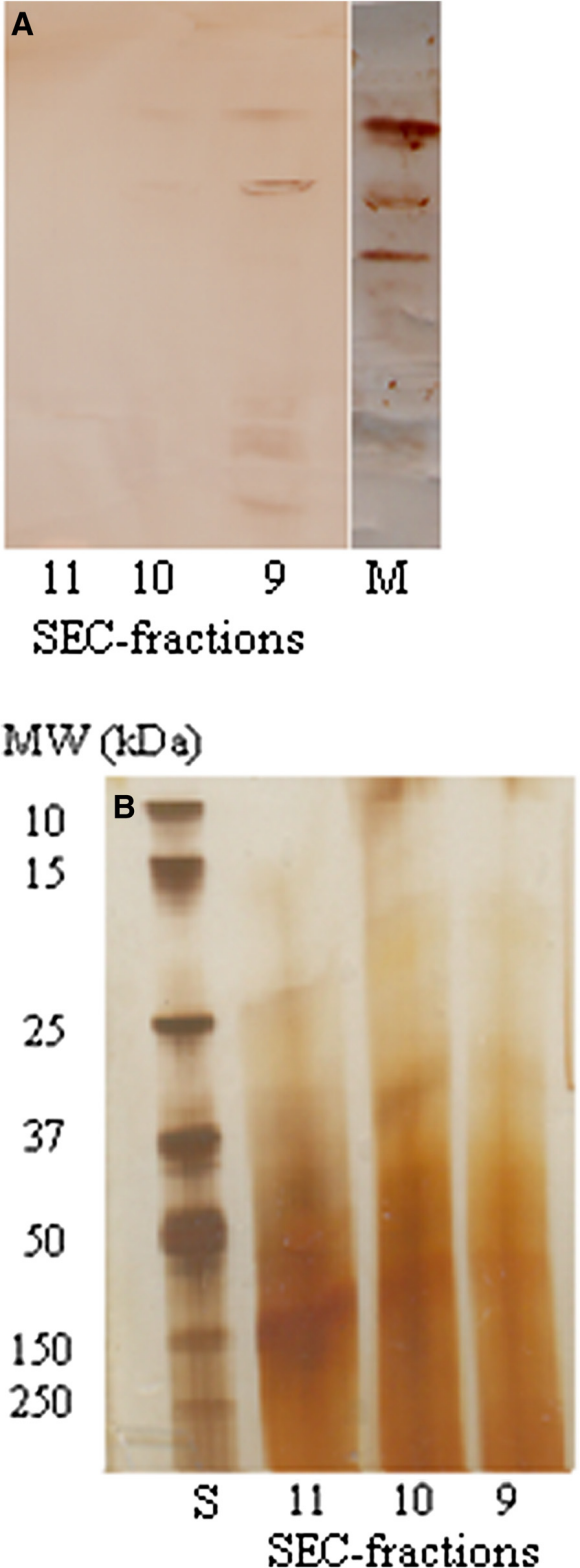
**Size exclusion chromatography of equine serum amyloid A.** Detection of serum amyloid A (SAA) isoforms and assessment of purity of SAA separated by size exclusion chromatography of freeze-dried equine acute phase serum dissolved in Milli-Q water. **A**: Immunostained isoforms of SAA after isoelectric focusing of size exclusion chromatography fractions 9–11. Note the incomplete isoform pattern compared to the SAA detected in freeze-dried serum dissolved in Milli-Q water (M, Figure 2A, lane 3), and note the detection of SAA in fraction 9, corresponding to a molecular weight of 237 kDa. No SAA was detected in the other 41 fractions. **B**: Silverstained gel following sodium dodecyl sulphate polyacryl amide gel electrophoresis (SDS-PAGE) for the assessment of presence of non-SAA impurities in the fraction analyzed in A. The molecular weights (MW) of the BioRad standard (S) are given to the left.

**Figure 5:**
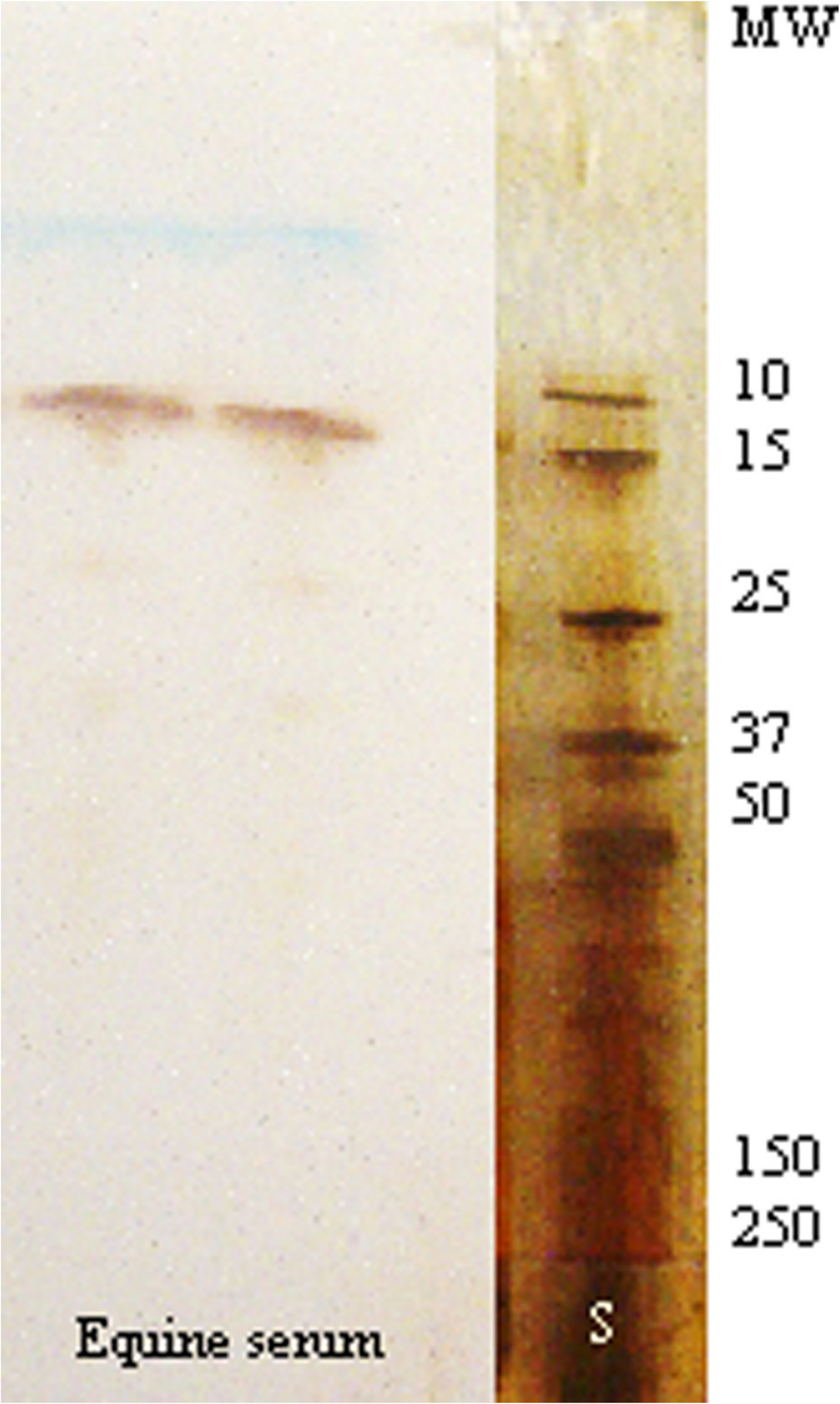
**Molecular weight of equine serum amyloid A.** Serum amyloid A (SAA) detected by immunostaining, following sodium dodecyl sulphate polyacryl amide gel electrophoresis (SDS-PAGE). As expected, the molecular weight of SAA was estimated to be 10–15 kDa. The molecular weights (MW) of the BioRad standard are given to the right (S). The standard lane was separated prior to blotting and stained with silver nitrate.

Forty-two fractions were obtained by FPLC-SEC of freeze dried serum diluted in Milli-Q water (Figure 1). SAA was detected in fraction 9 of the SEC (Figure 4), corresponding to an estimated molecular weight of 237 kDa, even though the molecular weight of SAA in equine serum was confirmed to be 10–15 kDa through SDS-PAGE (Figure 5). The isoform pattern of SAA in the FPLC-SEC-fraction (Figure 4) seemed to be incomplete compared to the material injected to the column (the supernatant obtained by the suspension of freeze-dried serum in Milli-Q water, Figure 2, lane 3), and several other proteins were detected in the SDS-PAGE analysis of the fraction, showing impurity of the separated SAA (Figure 4). No SAA was observed in the remaining 41 FPLC fractions.

**Figure 6:**
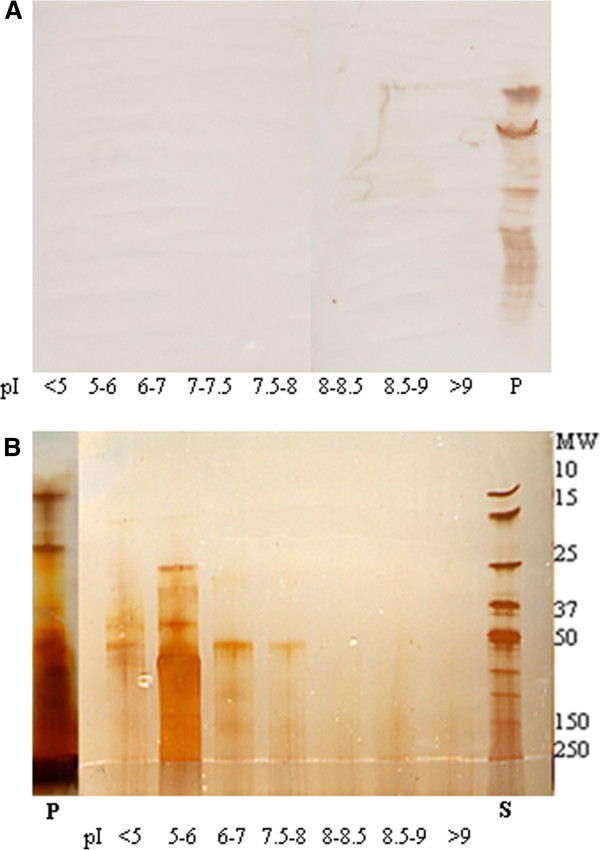
**Preparative isoelectric focusing of equine serum amyloid A.** Detection of serum amyloid A (SAA) isoforms and assessment of purity of SAA separated by preparative isoelectric focusing (IEF) of freeze-dried equine acute phase serum dissolved in 8 M urea. **A**: Immunostained isoforms of SAA after isoelectric focusing of concentrated preparative IEF fractions with pH of <5, 5–6, 6–7, 7–7.5, 7.5-8, 8–8.5, 8.5-9, >9. A visible precipitate was seen in the application chamber (pI 5–6) after the preparative isoelectric focusing, and was subsequently dissolved in 8 M urea. Note that SAA was only detected in this precipitate (P). **B**: Silverstained gel following sodium dodecyl sulphate polyacrylamide gel electrophoresis (SDS-PAGE) of equine serum proteins separated in 7 of the pI-intervals by preparative isoelectric focusing. Molecular weights (MW) of the Bio-Rad standard (S) are given to the right. Note that several serum proteins were detected in the precipitate described above (P).

No SAA was detected in fractions of preparative IEF (Figure 6A), even though protein migration was documented, especially of serum proteins with acidic isoelectric properties (Figure 6B). SAA was detected in a precipitate of several serum proteins formed on the gel surface facing chamber 2, in which sample material was applied (Figure 6).

## Discussion

Equine SAA seemed to have more hydrophilic properties than previously believed, and even though effective purification of SAA could not be achieved with the methods used in the present study, the results increase the knowledge about the biochemical properties and potentials for purification of equine SAA. Thus, the present results could be of importance in the development of protocols for production of species-specific material for calibration of diagnostic SAA assays, thus facilitating precise measurements of species-specific SAA.

Solubility of SAA in urea and various buffers containing phosphate or TRIS has previously been demonstrated [35], but to the author’s knowledge, this is the first report suggesting solubility of SAA in water, thus suggesting more hydrophilic properties of equine SAA than previously reported [5], similar to recent findings in pigs [29]. The solvents were chosen to facilitate isolation of SAA from other serum components based on the amphipathic properties of SAA: 2-propanol was chosen to dissolve SAA and other lipophilic substances, without dissolving hydrophilic serum components, Milli-Q-water was chosen to dissolve hydrophilic substances, without dissolving SAA and other lipophilic serum components, and 8M urea was chosen because of its chaothrophic properties and because it has been a useful media for analytical procedures of SAA detection [6,30,31]. Consequently, the dissolution of SAA in 70% 2-propanol and 8 M urea was expected. More isoforms and more intense bands of SAA were detected in 8 M urea, suggesting both quantitative and qualitative better solubility in this solvent, even though additional studies are needed to quantify the solubility and to investigate the maximal solubility in different solvents. The detection of SAA in freeze dried serum dissolved in Milli-Q water, however, suggests more hydrophilic properties than previously believed, or suggests interactions between SAA and other serum substances with more hydrophilic properties. Hydrophilic properties of SAA or SAA complexes, could also explain the failure to extract SAA by SFE, as lipophilic and ampophilic/lipophilic substances are expected to be extractable by this procedure, as previously observed for different drugs [36]. Additional studies are needed to investigate whether the biochemical properties of equine SAA is of similar nature as the recently observed properties demonstrated in pigs [29], but unlike in porcine SAA purification [29], HIC has previously been shown to be useful as an initial step for purification of equine SAA [5]. Thus, hydrophilic properties of SAA complexes seem to be a more probable explanation of the results than hydrophilic properties of the SAA molecule itself. Further, SAA precipitated during preparative IEF, when other serum components were separated. Other serum components seem, consequently, to be necessary in order to keep SAA solubilized under hydrophilic conditions, as also suggested previously [13]. Higher concentrations of urea would probably facilitate the separation of SAA isoforms by preparative IEF [35], thus avoiding the observed precipitation. However, the conditions used in the present study could potentially be useful as an early purification step separating proteins with acidic pI values. Such substances could for instance be albumin (pI 4.6-4.7 [37]), which has been shown to be one of the potential contaminants during SAA purifications [11].

Aggregation of lipoproteins with other serum proteins is commonly observed [15], and was further confirmed by the results of the FPLC-SEC, since SAA was separated with a higher apparent molecular weight than expected. The isoform pattern obtained by detection of SAA in the FPLC-SEC fraction was, however, incomplete and as the lacking isoforms were not detected in other fractions either, precipitation in the column, as observed after preparative IEF, or a partly binding to the column during the chromatography could not be ruled out. Repeated gel filtering has previously been used for purification of equine SAA [5,38], but because of many impurities observed in this and other studies [14], and because of the apparent loss of SAA isoforms during the FPLC-SEC in the present study this method seems to be less useful for purifications aiming to obtain calibration material.

In conclusion, our study indicated unexpected hydrophilic properties of equine SAA, as SAA could be detected following dissolution of lyophilized acute phase serum in Milli-Q water and as SAA was not extracted by SFE. Further studies are, however, needed to explore the nature of possible interactions with other serum components increasing the hydrophilic properties of SAA, and the significance of these unexpected biochemical properties. Our attempt to purify equine SAA was unsuccessful, but the results, however, contribute to the knowledge about the biochemical properties of SAA, and can consequently be of importance in future studies of SAA’s properties and purification.

## Abbreviations

FPLC: Fast protein liquid chromatography; IEF: Isoelectric focusing; SAA: Serum amyloid A; SDS-PAGE: Sodium dodecyl sulphate polyacryl amide gel electrophoresis; SEC: Size exclusive chromatography; SFE: Supercritical fluid extraction.

## Competing interests

The authors declare that they have no competing interests.

## Authors’ contributions

All authors were included in designing the framework of the study. MBC carried out the solubility and purification procedures and drafted the manuscript. JCS conceived the procedures of the study and helped to draft the manuscript, and SJ and MKH participated in the design of the study and helped to draft the manuscript. All authors approved the final manuscript.

## References

[B1] Eckersall PD Bell R Acute phase proteins: Biomarkers of infection and inflammation in veterinary medicine Vet J 2010 185 23 27 10.1016/j.tvjl.2010.04.009 20621712

[B2] Kjelgaard-Hansen M Jacobsen S Assay Validation and Diagnostic Applications of Major Acute-phase Protein Testing in Companion Animals Clin Lab Med 2011 31 51 70 10.1016/j.cll.2010.10.002 21295722

[B3] Jacobsen S Andersen P The acute phase protein serum amyloid A (SAA) as a marker of inflammation in horses Eq Vet Education 2007 19 38 46

[B4] Jacobsen S Jensen JC Frei S Jensen AL Thoefner MB Use of serum amyloid A and other acute phase reactants to monitor the inflammatory response after castration in horses: a field study Eq Vet J 2005 37 552 556 10.2746/042516405775314853 16295934

[B5] Hulten C Sletten K Bruun C Marhaug G The acute phase serum amyloid A protein (SAA) in the horse: isolation and characterization of three isoforms Vet Immunol Immunopathol 1997 57 215 227 10.1016/S0165-2427(97)00021-4 9261960

[B6] Jacobsen S Niewold T Halling-Thomsen M Nanni S Olsen E Lindegaard C Andersen P Serum amyloid A isoforms in serum and synovial fluid in horses with lipopolysaccharide-induced arthritis Vet Immunol Immunopathol 2006 110 325 330 10.1016/j.vetimm.2005.10.012 16337010

[B7] Bausserman L Herbert P Forte T Klausner R McAdam K Osborne J Rosseneu M Interaction of the Serum Amyloid a Proteins with Phospholipid J Biol Chem 1983 258 681 688 6411718

[B8] Benditt E Eriksen N Amyloid protein SAA is associated with high density lipoprotein from human serum Proc Nat Acad Sci USA 1977 74 4025 4028 10.1073/pnas.74.9.4025 198813 PMC431828

[B9] Carpintero R Pineiro M Andres M Iturralde M Alava M Heegaard P Jobert J Madec F Lampreave F The concentration of apolipoprotein A-I decreases during experimentally induced acute-phase processes in pigs Infect Immun 2005 73 3184 3187 10.1128/IAI.73.5.3184-3187.2005 15845530 PMC1087351

[B10] Ducret A Bruun C Bures E Marhaug G Husby G Aebersold R Characterization of human serum amyloid A protein isoforms separated by two-dimensional electrophoresis by liquid chromatography electrospray ionization tandem mass spectrometry Electrophoresis 1996 17 866 876 10.1002/elps.1150170508 8783012

[B11] Marhaug G Husby G Characterization of human amyloid-related protein SAA as a polymorphic protein: association with albumin and prealbumin in serum Clin Exp Immunol 1981 45 97 106 7307348 PMC1537261

[B12] Hocke G Kaffarnik H Munscher G Steinmetz A Purification of Human Serum Amyloid-a by Anion-Exchange Fast Protein Liquid-Chromatography J Chromatogr Biomed Appl 1990 526 203 209 10.1016/s0378-4347(00)82499-8 2341533

[B13] Ham D Karska-Wysocki B Purification and separation of hydrophobic serum amyloid a precursor isoforms by a one-step preparative method J Immunol Methods 2005 303 11 18 10.1016/j.jim.2005.05.013 16039662

[B14] Hulten C Tulamo RM Suominen MM Burvall K Marhaug G Forsberg M A non-competitive chemiluminescence enzyme immunoassay for the equine acute phase protein serum amyloid A (SAA) – a clinically useful inflammatory marker in the horse Vet Immunol Immunopathol 1999 68 267 281 10.1016/S0165-2427(99)00027-6 10438325

[B15] Peeters H Peeters H The challenge of the lipoprotein molecule Proceedings of the NATO Advanced Study Institute on the Lipoprotein Molecule: 8-20 May 1977; Belgium 1978 New York: Plenum Press 3 6

[B16] Yamada T Serum amyloid A (SAA): a concise review of biology, assay methods and clinical usefulness Clin Chem Lab Med 1999 37 381 388 10369107 10.1515/CCLM.1999.063

[B17] Havel RJ Eder HA Bragden JH The distribution and chemical composition of ultracentrifugally separated lipoproteins in human serum J Clin Invest 1955 34 1345 1353 10.1172/JCI103182 13252080 PMC438705

[B18] Raynes J McAdam K Purification of Serum Amyloid-a and Other High-Density Apolipoproteins by Hydrophobic Interaction Chromatography Anal Biochem 1988 173 116 124 10.1016/0003-2697(88)90168-6 3142296

[B19] Soler L Gutiérrez A Martinez-Subiela S Ceron JJ Development and validation of a novel competitive ELISA for the detection of serum amyloid A in pigs Vet J 2011 190 e7 e11 10.1016/j.tvjl.2011.02.016 21421332

[B20] Hansen AE Schaap MK Kjelgaard-Hansen M Evaluation of a commercially available human serum amyloid A (SAA) turbidimetric immunoassay for determination of feline SAA concentration Vet Res Commun 2006 30 863 872 10.1007/s11259-006-3373-6 17139536 PMC7089358

[B21] Pepys M Baltz M Tennent G Kent J Ousey J Rossdale P Serum Amyloid a Protein (Saa) in Horses - Objective Measurement of the Acute Phase Response Eq Vet J 1989 21 106 109 10.1111/j.2042-3306.1989.tb02108.x 2539996

[B22] Christensen M Jacobsen S Ichiyanagi T Kjelgaard-Hansen M Evaluation of an automated assay based on monoclonal anti-human serum amyloid A (SAA) antibodies for measurement of canine, feline, and equine SAA Vet J 2012 194 332 337 10.1016/j.tvjl.2012.05.007 22704135

[B23] Jacobsen S Kjelgaard-Hansen M Evaluation of a commercially available apparatus for measuring the acute phase protein serum amyloid A in horses Vet Rec 2008 163 327 330 10.1136/vr.163.11.327 18791207

[B24] Jacobsen S Kjelgaard-Hansen M Hagbard Petersen H Jensen AL Evaluation of a commercially available human serum amyloid A (SAA) turbidometric immunoassay for determination of equine SAA concentrations Vet J 2006 172 315 319 10.1016/j.tvjl.2005.04.021 15950503

[B25] Eckersall P Duthie S Toussaint M Gruys E Heegaard P Alava M Lipperheide C Madec F Standardization of diagnostic assays for animal acute phase proteins Adv Vet Med 1999 41 643 655 9890051 10.1016/s0065-3519(99)80050-0

[B26] Kjelgaard-Hansen M Comments on measurement of C-reactive protein in dogs Vet Clin Path 2010 39 402 403 10.1111/j.1939-165X.2010.00276.x 21198730

[B27] Malle E Hess H Munscher G Knipping G Steinmetz A Purification of Serum Amyloid-a and its Isoforms from Human Plasma by Hydrophobic Interaction Chromatography and Preparative Isoelectric-Focusing Electrophoresis 1992 13 422 428 10.1002/elps.1150130189 1425555

[B28] Strachan A De Beer FC Van der Westhuyzen D Coetzee G Identification of 3 Isoform Patterns of Human-Serum Amyloid-a Protein Biochem J 1988 250 203 207 3355511 10.1042/bj2500203 PMC1148833

[B29] Soler L Molenaar A Merola N Eckersall PD Gutiérrez A Ceron JJ Mulero V Niewold T Why working with porcine circulating serum amyloid A is a pig of a job J Theor Biol 2013 317 119 125 23073471 10.1016/j.jtbi.2012.10.011

[B30] Jacobsen S Niewold T Kornalijnslijper E Toussaint M Gruys E Kinetics of local and systemic isoforms of serum amyloid A in bovine mastitic milk Vet Immunol Immunopathol 2005 104 21 31 10.1016/j.vetimm.2004.09.031 15661328

[B31] Kjelgaard-Hansen M Christensen MB Lee MH Jensen AL Jacobsen S Serum amyloid A isoforms in serum and synovial fluid from spontaneously diseased dogs with joint diseases or other conditions Vet Immunol Immunopathol 2007 117 296 301 10.1016/j.vetimm.2007.03.008 17451811

[B32] Chevallet M Luche S Rabilloud T Silver staining of proteins in polyacrylamide gels Nat Protoc 2006 1 1852 1858 10.1038/nprot.2006.288 17487168 PMC1971133

[B33] Righetti P Wenisch E Faupel M Preparative Protein-Purification in a Multi-Compartment Electrolyzer with Immobiline Membranes J Chromatogr 1989 475 293 309 10.1016/S0021-9673(01)89684-9

[B34] Righetti P Bossi A Wenisch E Orsini G Protein purification in multicompartment electrolyzers with isoelectric membranes J Chrom B 1997 699 105 115 10.1016/S0378-4347(97)00156-4 9392371

[B35] Strachan A Shephard E Bellstedt DU Coetzee G van der Westhuyzen D De Beer F Human serum amyloid A protein. Behavior in aqeous and urea-containing solutions and antibody production Biochem J 1989 263 365 370 2597108 10.1042/bj2630365 PMC1133438

[B36] Klimes J Sochor J Kriz J A study of the conditions of the supercritical fluid extraction in the analysis of selected anti-inflammatory drugs in plasma Farmaco 2002 57 117 122 10.1016/S0014-827X(01)01182-X 11902653

[B37] Chaiyasut C Tsuda T Isoelectric points estimation of proteins by electroosmotic flow: pH relationship using physically adsorbed proteins on silica gel Chromatography 2001 22 91 95

[B38] Nunokawa Y Isolation, characterization and quantitative analysis of serum amyloid A protein from horses Jap J Vet Res 1992 40 49

